# Comparing the Behavioral Impacts of Heavy Metals and Rare Earth Elements on Black Soldier Fly (*Hermetia illucens*) Larvae

**DOI:** 10.3390/toxics14080729

**Published:** 2026-08-17

**Authors:** Muhammad Baqir Khan, Minh-Quan Tran, Petrus Siregar, Szu-Chieh Wang, Ming-Der Lin, Chung-Der Hsiao

**Affiliations:** 1 Department of Biosciences, Chung Yuan Christian University, No. 200, Zhongbei Rd., Zhongli Dist., Taoyuan 320314, Taiwan; baqirkhanmmc@gmail.com (M.B.K.); minhquan0141@gmail.com (M.-Q.T.); siregar.petrus27@gmail.com (P.S.); 2 Department of Biomedical Engineering, Chung Yuan Christian University, No. 200, Zhongbei Rd., Zhongli Dist., Taoyuan 320314, Taiwan; 3 Department of Molecular Biology and Human Genetics, Tzu Chi University, Hualien 970374, Taiwan; scwang2023@gmail.com; 4 Center for Herbal Medicine and Natural Products Research, Tzu Chi University, Hualien 970374, Taiwan; 5 Department of Biotechnology, Kaohsiung Medical University, Kaohsiung City 80708, Taiwan; 6 Department of Chemistry, Chung Yuan Christian University, Taoyuan 320314, Taiwan; 7 Research Center of Aquatic Toxicology and Pharmacology, Chung Yuan Christian University, Taoyuan 320314, Taiwan

**Keywords:** black soldier fly, heavy metal, rare earth element, behavior, phenomic

## Abstract

Heavy metals (HMs) and rare earth elements (REEs) increasingly co-occur in environmental waste streams and soils, yet their comparative neurotoxic mechanisms and sublethal effects on invertebrate decomposers remain poorly understood. This study used black soldier fly larvae (BSFL, *Hermetia illucens*) to perform a comparative behavioral and transcriptomic assessment of 23 HMs and 16 REEs across two acute exposure concentrations. High-throughput video tracking and phenomic analysis showed HMs produced broader disruption than REEs, with low-concentration HMs inducing locomotor suppression, thigmotaxis, reduced fractal dimension and entropy, progressing to severe motor inhibition and rigid low-entropy states. In contrast, REEs showed a biphasic profile, shifting from selective locomotor suppression with preserved organization at low concentrations to hyperactive, fragmented, high-entropy movement with increased thigmotaxis at high concentrations. PCA and hierarchical clustering integrated endpoints into four neurobehavioral fingerprints segregating metal class and concentration, with partial overlap of high-concentration REEs with HMs along a shared high-toxicity axis. Transcriptomic profiling showed that cobalt as a representative HM activated DNA damage and cell-cycle pathways, perturbed energy signaling, and suppressed neuroactive ligand–receptor interaction, whereas samarium as a representative REE downregulated xenobiotic metabolism, oxidative phosphorylation, glutathione metabolism, and synaptic vesicle cycling. These findings demonstrate distinct concentration-dependent neurotoxic modes of action for HMs and REEs and establish BSFL behavioral phenomics integrated with transcriptomics as a mechanistically informative platform for ecological risk assessment in contaminated waste systems.

## 1. Introduction

Heavy metals and rare earth elements (REEs) are significant environmental pollutants due to their widespread industrial application, environmental persistence, and potential toxicity [1,2]. HMs such as cadmium (Cd), lead (Pb), chromium (Cr), and nickel (Ni) are released into ecosystems through natural processes and human activities including mining, industrial emissions, agriculture, and waste disposal [3,4]. These are non-biodegradable and bioaccumulative elements, they persist in soil, water, and organisms, posing serious risks to ecosystems and human health. Exposure to these toxicants can rapidly induce neurobehavioral impairment, oxidative stress, and cellular toxicity, making them a major focus of toxicology recently [5,6]. In recent decades, the extraction and use of REEs have increased substantially due to their importance in modern technologies such as electronics, renewable energy systems, and defense applications [7,8]. This rapid expansion has raised concerns regarding their environmental release and potential toxicological effects [9,10]. Although REEs share certain chemical characteristics with heavy metals, their toxicity mechanisms and long-term ecological impacts remain poorly understood [11]. Importantly, HMs and REEs often co-occur in contaminated environments, resulting in complex mixed exposures with additive or synergistic effects. This complicates ecological risk assessment and highlights the need for integrated, high-throughput, and mechanism-based toxicological evaluation [12].

The Black Soldier Fly (*Hermetia illucens*), particularly its larval stage (BSFL), is a valuable model organism for toxicity and environmental studies due to its robust biology and ecological relevance [13]. BSFL exhibit rapid growth, broad environmental tolerance, and strong bioaccumulative capacity, enabling sensitive responses to toxicant exposure [14,15]. They have gained attention for their role in organic waste decomposition and nutrient recycling, providing a realistic ecological context for contaminant assessment [16,17]. Their ability to thrive on diverse substrates has expanded their application in sustainable agriculture, animal feed production, and environmental toxicology [18,19,20]. BSFL can accumulate and respond to diverse contaminants, including pesticides, heavy metals, and mycotoxins; for example, malathion induces oxidative stress across developmental stages, demonstrating their utility in toxicity assessment [21]. Furthermore, bioaccumulation and microbiota-mediated detoxification highlight BSFL as promising bioindicators for environmental risk assessment [20,22]. Collectively, these features suggest that BSFL are particularly suitable for studying persistent environmental pollutants, including heavy metals (HMs) and rare earth elements (REEs) [23]. Importantly, toxicant-induced effects can be rapidly and sensitively quantified through BSFL behavioral responses using high-throughput screening assays, highlighting their value as an efficient and sustainable model for environmental toxicity research.

Despite extensive research on emerging pollutants, the behavioral toxicity of heavy metals (HMs) and rare earth elements (REEs) remains poorly understood. Behavioral endpoints, including locomotion and movement complexity, serve as sensitive indicators of sublethal toxicity [24]. BSFL, with their high-throughput capacity and quantifiable behavioral responses, are well suited to comparative toxicity studies. This study evaluated the relative toxicity of HMs and REEs, their effects on BSFL, and their potential as novel bioindicators [24]. This approach addresses knowledge gaps in HMs and REEs toxicity while highlighting BSFL behavioral assays for environmental risk assessment. Behavioral responses are increasingly recognized as sensitive and ecologically relevant endpoints, providing early warning of sublethal stress before physiological or lethal effects become evident [25]. BSFL show measurable changes in movement, such as reduced crawling speed after acrylamide exposure, indicating neurotoxic effects [26]. Such alterations reflect nervous system function and energy status, enabling early detection of toxicity. Alterations in movement patterns, substrate preference, and activity levels reflect nervous system function and energy reserves, allowing early detection of neurotoxicity, metabolic disruption, or compromised health [26]. Integrating behavioral endpoints enhances the sensitivity and ecological relevance of BSFL-based toxicity assessments, particularly in waste bioconversion systems where they play key ecological and economic roles [27].

**Figure 1:**
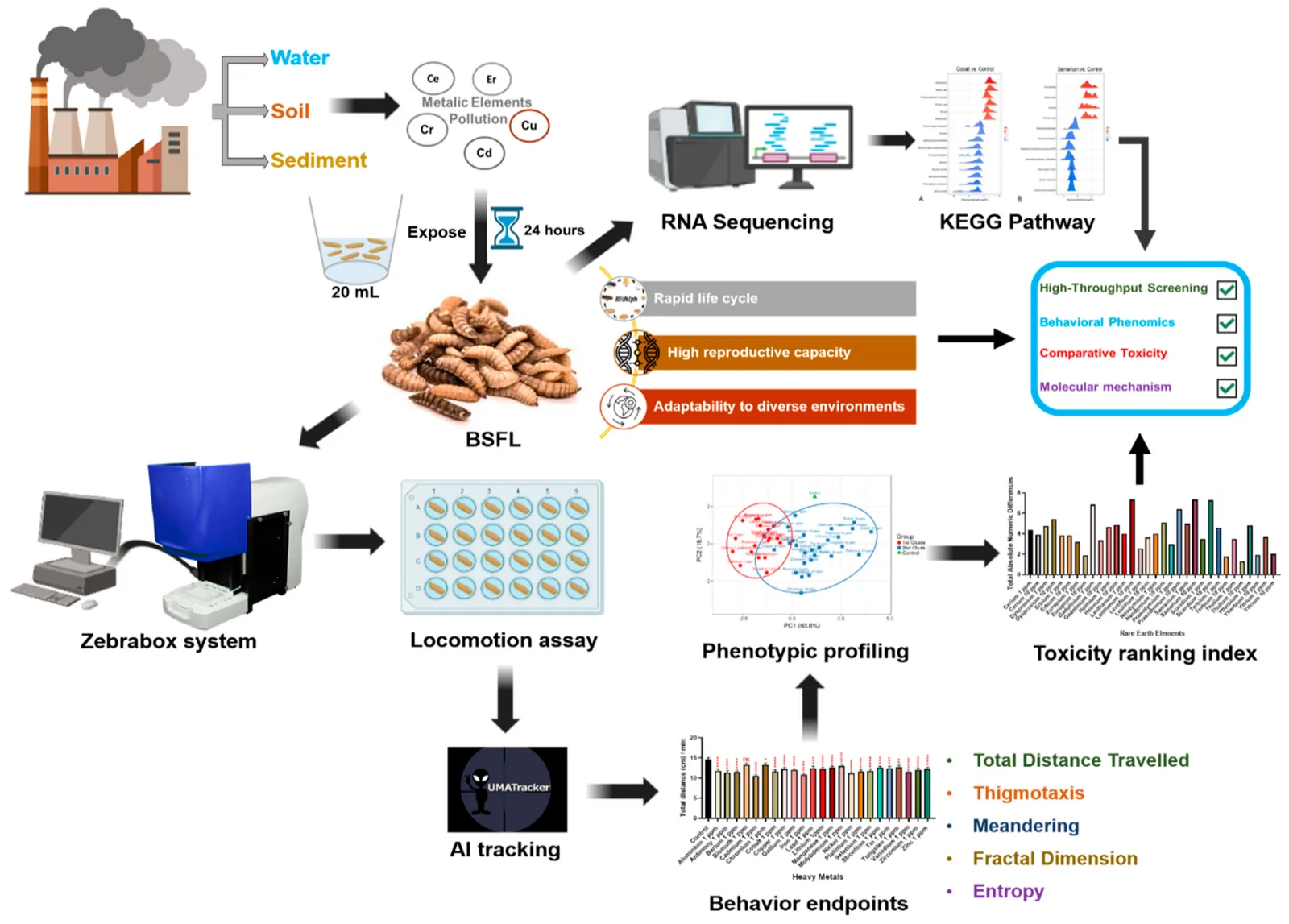
Integrated workflow for high-throughput ecotoxicological assessment of metal-contaminated environments using BSFL. Industrial activities release mixed metallic elements into water, soil, and sediment, which serve as exposure matrices for larvae under controlled conditions. BSFL are used as a robust invertebrate model due to their rapid development, high adaptability, and suitability for toxicological screening. Following exposure, a multi-level analytical framework is applied. At the organismal level, automated behavioral phenotyping quantifies locomotor and exploratory parameters such as distance traveled, thigmotaxis, meandering, fractal dimension, and entropy, capturing neurobehavioral alterations induced by metal stress. Multivariate analyses including PCA and clustering distinguish exposure groups and visualize phenotypic separation, supported by comparative bar plots. At the molecular level, transcriptomic profiling identifies differentially expressed genes and associated pathways linked to metal-induced stress responses. Together, this integrated pipeline combines phenotypic, statistical, and molecular readouts to enable scalable, mechanistically informed assessment of metal toxicity in environmental systems (the figure was created by using biorender.com).

In this study, we systematically compared the sublethal behavioral toxicity of 23 heavy metals (metalloids) (HMs) and 16 rare earth elements (REEs) in black soldier fly larvae (BSFL), an ecologically relevant and high-throughput invertebrate model. This work is novel in providing the first large-scale, side-by-side behavioral profiling of HMs and REEs in BSFL by integrating quantitative behavioral endpoints, including distance traveled, entropy, fractal dimension, meandering, and thigmotaxis, with multivariate phenomic analyses and transcriptomic profiling (Figure 1). To achieve these objectives, we established a sensitive video-tracking behavioral assay to quantify locomotor responses following metal exposure and combined behavioral phenotyping with transcriptomic analysis to investigate the molecular mechanisms underlying the observed behavioral alterations. This integrated approach enables the identification of relative toxicity patterns, distinguishes metal-specific neurobehavioral signatures, and evaluates whether REEs pose ecological risks comparable to conventional HMs. Ultimately, the findings advance the application of BSFL as a novel bioindicator for metallic contamination and contribute to the development of more efficient environmental risk assessment frameworks and sustainable waste bioconversion strategies.

## 2. Materials and Methods

### 2.1. Black Soldier Fly Larvae Maintenance

In this study, BSF larvae were reared under controlled laboratory conditions, with temperature (27–30 °C), relative humidity (60–70%), and a 12:12 h light–dark cycle, as reported in previous studies [28]. Vegetable waste mixed with moistened wheat bran was used as the standard substrate, maintained at 65–70% moisture to support growth and digestion. The substrate was refreshed every 2–3 days to prevent microbial contamination and maintain hygiene [29]. Larvae were kept in plastic containers (30 × 20 × 10 cm) with covered lids to allow proper aeration, and density for behavioral assessments was maintained at 5–10 larvae per gram of substrate [30]. Development, survival, and signs of stress were monitored daily, and unfit or dead larvae were promptly removed [31]. All tools and surfaces were sterilized daily with hot water or 70% ethanol, and personnel used PPE, following institutional biosafety protocols for safe waste disposal.

### 2.2. Heavy Metals and Rare Earth Elements Exposure

**Figure 2:**
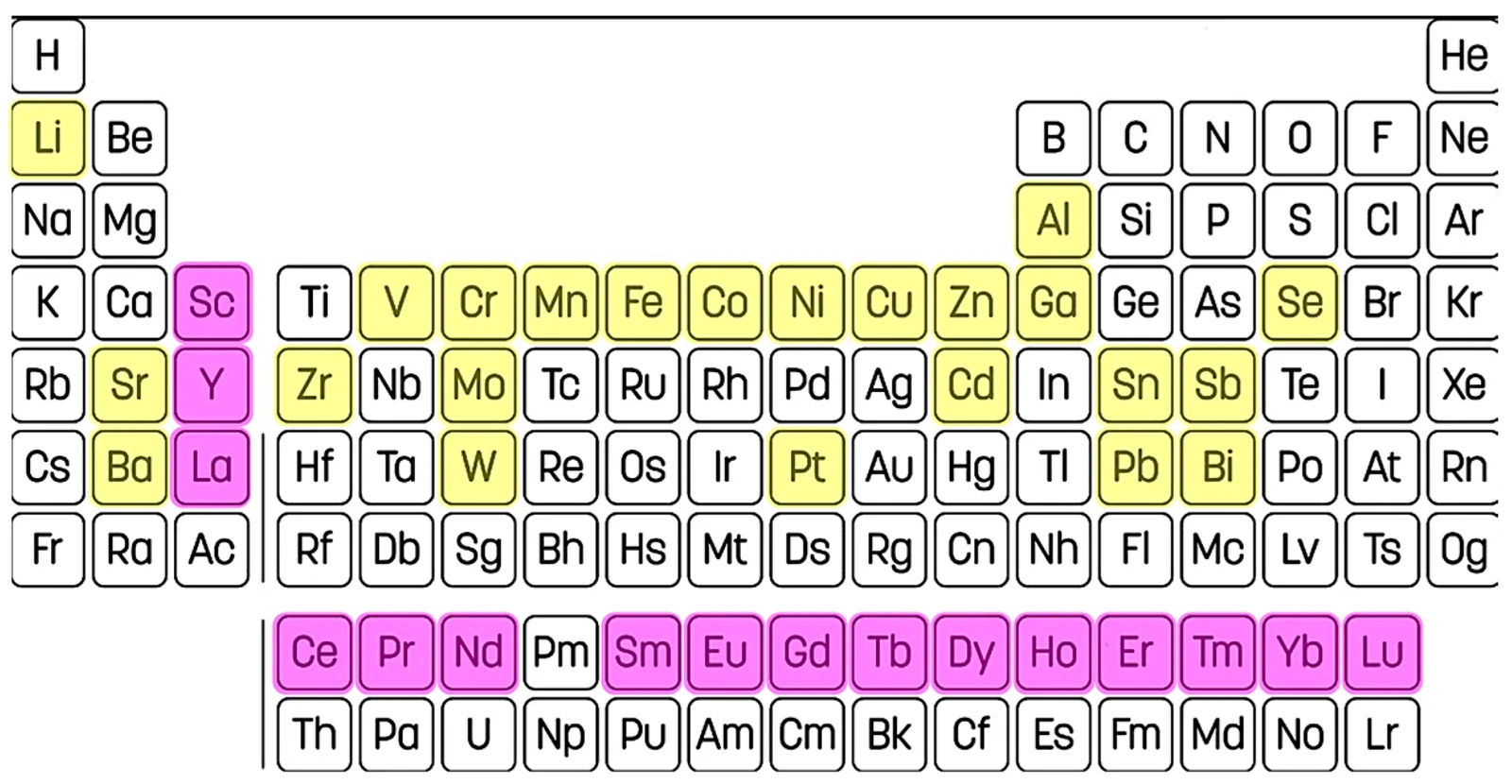
Metallic toxicants tested in this study are distinguished by color, with yellow representing heavy metals (metalloids) (HMs) and pink representing rare earth elements (REEs).

BSF larvae were exposed to heavy metals using a glass exposure setup with LED illumination. Stock solutions (5000 ppm) were prepared and serially diluted to 1 and 10 ppm (mg/L), representing environmentally relevant concentrations reported previously [32]. An initial large-scale screening was conducted for 23 heavy metals (Table S1) and all 16 rare earth elements (Table S2). For each treatment, 20 mL of working solution was prepared in double-distilled water (ddH_2_O), and larvae were carefully transferred from the rearing containers into the exposure glasses for 24 h. Throughout the exposure period, survival was assessed immediately before behavioral recording by counting live and dead larvae in each treatment group. Larvae showing no visible movement and no response to gentle mechanical stimulation were considered dead. No mortality was observed in any treatment group at either 1 or 10 ppm. Following exposure, locomotor activity was recorded using the ZebraBox ViewPoint system. Experiments were performed in three independent biological replicates for each toxicant (Figure 2), with 24 larvae per replicate (total *n* = 72).

### 2.3. Video Recording Using ZebraBox Viewpoint System

BSF larvae were exposed to heavy metals for 24 h, after which their behavioral responses were recorded to assess sublethal effects. Videos were captured using a ZebraBox viewpoint system (Viewpoint Life Sciences, Lyon, France) [24,33]. Prior to recording, larvae underwent a 5-min acclimation period in a home-made acrylic plate (13 × 8 cm) to familiarize them with the environment, and the light intensity was set to the maximum level. The acrylic plate contained 24 individual wells, with one larva per well, allowing simultaneous observation of 24 larvae in a single recording session (Figure 1). Recording duration was 10 min per session, providing sufficient data to capture locomotor and exploratory behaviors following exposure.

### 2.4. The Locomotion Tracking Using UMATracker

After video acquisition using the ZebraBox viewpoint system, recordings were imported into UMATracker release-15, a behavior tracking software [34]. UMATracker has been widely applied for behavioral analyses in aquatic and semi-aquatic organisms, including color pattern analysis in freshwater crayfish, locomotor activity in freshwater shrimp, and zebrafish movement tracking, demonstrating its reliability as a behavioral tracking tool [35,36,37]. In this study, two core UMATracker modules were utilized: FilterGenerator and Tracking. The FilterGenerator, which serves as the preprocessing phase, was implemented using the visual programming environment Blockly to optimize tracking parameters. This step involved image thresholding, morphological operations for noise reduction, and region-of-interest (ROI) selection to eliminate background interference. Video frames were converted to grayscale and binarized by adjusting threshold values to effectively distinguish larvae from the background. Following preprocessing, the Tracking module was employed by defining essential parameters, including the number of individuals, threshold intensity, and minimum and maximum object size. UMATracker tracks the centroid of each larva rather than the anterior region, enabling accurate representation of movement trajectories. Upon completion, X and Y coordinate positions for each larva in every frame were exported as .csv files. These coordinates served as the primary dataset for calculating behavioral endpoints of BSFL locomotion and activity.

### 2.5. BSFL Behavioral Endpoints

Following the establishment of the behavioral recording protocol, six behavioral endpoints were extracted to characterize Black Soldier Fly Larvae (BSFL) movement: total distance traveled, average angular velocity, meandering, thigmotaxis, fractal dimension (FD), and entropy. Thigmotaxis was used to assess exploratory behavior and was calculated as the average distance of larvae from the central area, reflecting their tendency to remain close to boundaries during exploration [38]. Movement orientation was evaluated using average angular velocity and meandering. Angular velocity was calculated as the rate of directional change between consecutive time points, following approaches previously applied in zebrafish behavioral studies [39,40,41,42]. Meandering, defined as the ratio of total turning angle to distance traveled, was used to describe zigzag-like movement patterns. Movement complexity and predictability were further analyzed using fractal dimension (FD) and entropy. FD was employed to quantify the complexity of larval movement trajectories, while entropy measured the predictability and directional organization of movement patterns. The mathematical formulations for FD and entropy were adapted from established zebrafish-based behavioral analyses [43,44].

### 2.6. RNA-Seq and Gene Set Enrichment Analysis

BSFL were divided into three groups: (1) control, (2) cobalt exposure, and (3) samarium exposure. Because transcriptomic sequencing of all 39 metallic elements was beyond the scope of this study, one representative compound from each metal class was selected for mechanistic investigation. Cobalt was chosen from the heavy metals. It consistently ranked among the most behaviorally toxic compounds, whereas samarium was selected from the rare earth elements because it produced one of the strongest and most distinctive behavioral phenotypes. These compounds were therefore used as representative case studies to investigate molecular pathways underlying the contrasting behavioral profiles of HMs and REEs. Given the chemical diversity within both metal classes, the transcriptomic findings should be interpreted as mechanistic examples rather than universal molecular signatures.

Larvae were exposed under the same conditions as the behavioral assays and collected 24 h post exposure for RNA sequencing and pathway enrichment analysis. Three pooled RNA samples were prepared per group, each containing three larvae. Whole larvae were homogenized in RNAzol^®^ RT (100 mg/mL) using a Bullet Blender with magnetic beads. Following homogenization, 0.4 mL double-distilled water was added per 1 mL RNAzol^®^, incubated at room temperature for 15 min, and centrifuged at 12,000 rpm for 15 min to obtain the RNA-containing supernatant. RNA was precipitated with isopropanol, incubated for 10 min, centrifuged at 12,000 rpm for 10 min, washed three times with 75% ethanol, air-dried, and re-suspended in double-distilled water. RNA purity and concentration were assessed using OD_260/280_ measurements, and samples were stored at −20 °C until further analysis [45,46].

For RNA-seq, directed libraries were prepared using the TruSeq Stranded mRNA Library Prep Kit (Illumina, San Diego, CA, USA) according to the manufacturer’s protocol. Libraries were sequenced on the Illumina platform (paired-end 150 bp) by KIM FOREST Co., Ltd., New Taipei City, Taiwan. FASTQ files were quality-filtered using fastp (version 0.24.0) [47] and quasi-mapped to the *Hermetia illucens* reference transcriptome (GCA_905115235.1) using Salmon (version 1.10.3) [48] for transcript-level abundance estimation. Salmon output was imported into R using tximport, and gene-level counts were summarized prior to downstream analysis. Gene expression counts were analyzed using edgeR (v4.6.3). Raw read counts were normalized using the trimmed mean of M-values (TMM) method, and lowly expressed genes were filtered using the filterByExpr function with default parameters prior to model fitting. A generalized linear model (GLM) framework was constructed using experimental groups, and differential expression testing was performed using likelihood ratio tests (LRT). The biological coefficient of variation (BCV) was fixed at 0.2, a commonly used conservative estimate for insect transcriptomic studies, and contrast matrices were defined to compare all experimental groups [49,50]. To enable KEGG-based GSEA on the *H. illucens* genome, KO identifiers were assigned to each protein by prioritizing eggNOG-mapper annotations; proteins lacking eggNOG-mapper KO assignments were supplemented with KO identifiers from GhostKOALA. Protein-level KO assignments were subsequently converted to gene-level annotations by extracting protein_id-gene_id pairs from CDS features of the genome GTF annotation file and mapped to KEGG pathways using the KEGG pathway-to-KO relationship database. KEGG pathway gene sets with fewer than 8 or more than 500 annotated genes were excluded prior to enrichment testing. GSEA was performed using the fgsea package (v1.34.2) with 20,000 permutations. For each contrast, genes were ranked by signed effect size using the statistic sign(logFC) × √LR, where LR is the likelihood ratio test statistic. This metric emphasizes biologically consistent directional changes and stabilizes ranking compared to raw *p*-values. fgsea was executed with 20,000 permutations. Gene sets with FDR-adjusted *p*-values (padj) < 0.1 were considered significantly enriched. The raw RNA-seq data (FASTQ files) generated in this study have been deposited in the NCBI Sequence Read Archive (SRA) under BioProject accession number PRJNA 1467192.

### 2.7. Statistical Analysis

All statistical analyses and graphical presentations were performed using GraphPad Prism version 8.0.2 (GraphPad Software Inc., La Jolla, CA, USA). Behavioral endpoints among treatment groups were compared with the control group. Statistical evaluation was performed using the Kruskal–Wallis test (non-parametric one-way ANOVA) followed by Dunn’s multiple comparisons post hoc test. All results are presented as mean ± standard error of the mean (SEM). Statistical significance was defined as * *p* < 0.05, ** *p* < 0.01, *** *p* < 0.001, and **** *p* < 0.0001.

### 2.8. PCA, Hierarchical Clustering, and Toxicity Ranking

To enable comprehensive phenotypic profiling and reduce data dimensionality, Principal Component Analysis (PCA), hierarchical clustering, and toxicity ranking were performed after calculation of all behavioral endpoints [24,51]. Behavioral data were averaged for each treatment group, normalized relative to controls, compiled in Microsoft Excel, and exported as comma-separated values (.csv) files. PCA, a widely used projection-based dimensionality reduction technique, was applied to transform high-dimensional behavioral data into orthogonal components and visualize major variance patterns among treatments [52,53]. Hierarchical clustering and heatmap analyses were then conducted to identify behavioral alteration patterns induced by heavy metals (HMs) and rare earth elements (REEs).

Clustering and visualization were performed using the web-based ClustVis platform (https://biit.cs.ut.ee/clustvis, accessed on 26 January 2026) [53]. To quantify sublethal behavioral toxicity, heatmap-derived values for each metal were converted into numerical scores, and endpoint-specific responses were integrated to generate a composite toxicity index for each compound. Metals were subsequently ranked according to their cumulative index values, with higher scores indicating greater toxic potency. Detailed toxicity ranking calculations are provided in the Supplementary Materials (Tables S3 and S4). This quantitative phenomic approach enables systematic and hierarchical comparison of behavioral toxicity across multiple metals and is consistent with established methods for ranking multidimensional toxicity responses [54].

## 3. Results

### 3.1. Behavioral Alterations of Black Soldier Fly Larvae Following Acute Heavy Metal Exposure

**Figure 3:**
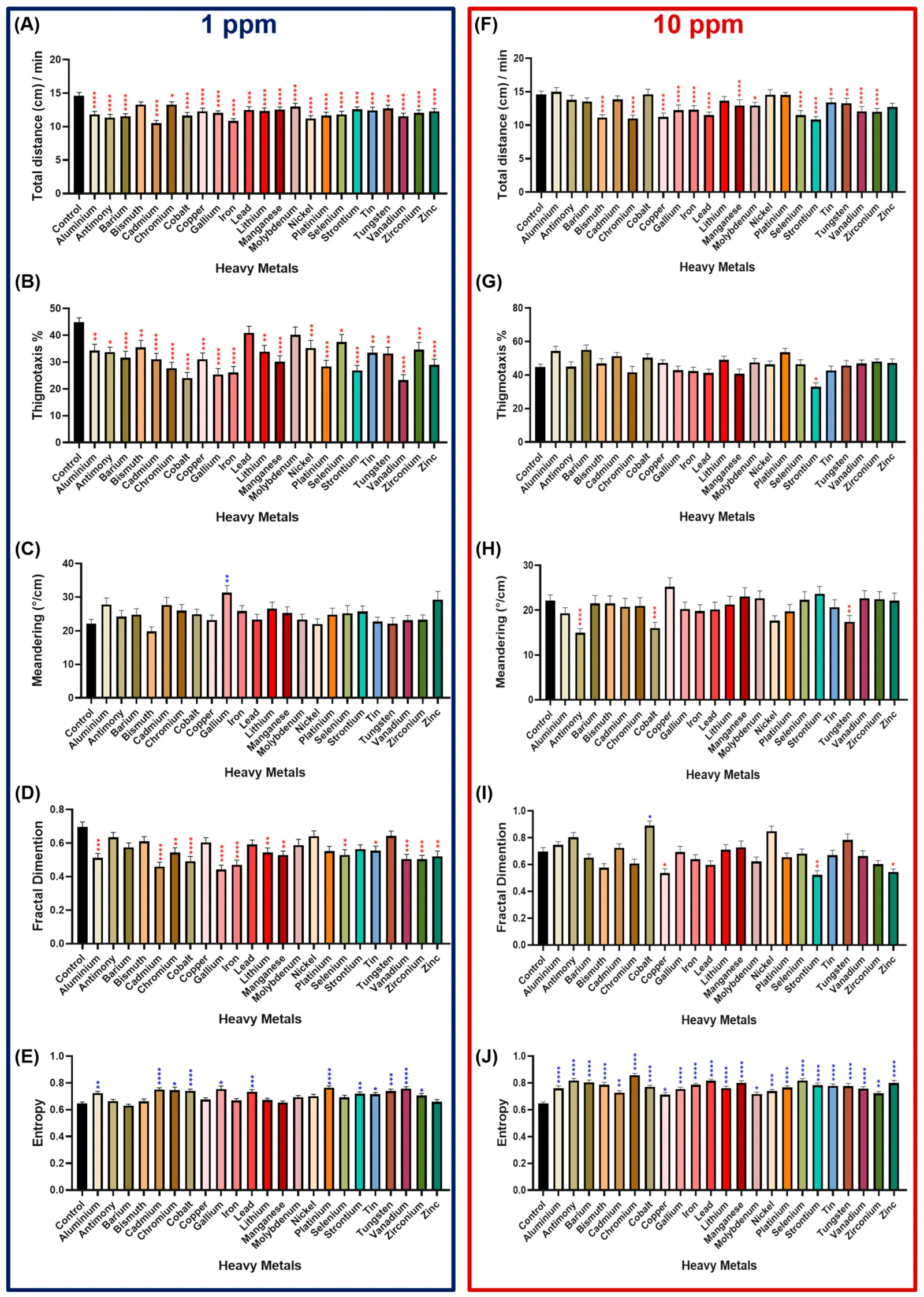
Black soldier fly (BSF) larvae were exposed to a panel of heavy metals for 24 h, and behavioral responses were quantified using five endpoints. For the 1 ppm exposure group, measured parameters included (**A**) total distance travelled, (**B**) thigmotaxis, (**C**) meandering, (**D**) fractal dimension, and (**E**) entropy. The same behavioral endpoints were assessed for larvae exposed to 10 ppm heavy metals: (**F**) total distance travelled, (**G**) thigmotaxis, (**H**) meandering, (**I**) fractal dimension, and (**J**) entropy. Data are presented as mean ± SEM and were analyzed using one-way ANOVA followed by Dunn’s multiple-comparison test. Statistical significance is indicated as * *p* < 0.05, ** *p* < 0.01, *** *p* < 0.001, and **** *p* < 0.0001 (*n* = 72).

A comprehensive behavioral screening characterized acute effects of 23 heavy metals on Black Soldier Fly (BSF) larvae after 24 h exposure at 1 ppm (Figure 3A–E), using locomotor and spatial endpoints including total distance travelled, thigmotaxis, meandering, fractal dimension, and entropy.

At low concentration 1 ppm, locomotor suppression was the most consistent response, with 22 of 23 metals significantly reducing total distance travelled; bismuth was the only exception (Figure 3A), indicating a broad reduction in motor output under sublethal exposure. Beyond reduced activity, metals induced clear changes in behavioral organization. Entropy increased in approximately half of the metals (Figure 3E), indicating increased movement unpredictability and neurobehavioral instability, while fractal dimension decreased in many metals (Figure 3D), reflecting reduced trajectory complexity and spatial restriction. Together, increased entropy with reduced fractal dimension indicates disorganized locomotion rather than simple hypoactivity. Spatial behavior was also altered, as most metals significantly increased thigmotaxis, consistent with anxiety-like or stress-related responses; lead and molybdenum were the only exceptions with no significant effect (Figure 3B). In contrast, meandering remained largely unchanged, with gallium as the sole metal causing a significant effect (Figure 3C), suggesting preserved directional stability at low concentration.

At higher concentration 10 ppm (24 h; Figure 3F–J), larvae exhibited a distinct high-concentration toxicity profile dominated by severe locomotor suppression, with nearly all metals significantly reducing total distance travelled (Figure 3F); some metals like aluminum, antimony, barium, and nickel showed no significant effect, and chromium produced only minimal changes. Unlike at low concentration, thigmotaxis remained unchanged across all metals (Figure 3G), indicating loss of behavioral flexibility rather than enhanced anxiety-like behavior. Directional movement showed metal-specific effects: antimony, cobalt, and tungsten significantly reduced meandering (straighter trajectories), and all other metals showed no effect (Figure 3H). Different from low concentration (1 ppm), fractal dimension responses were metal-specific, with only copper, strontium, and zinc decreasing trajectory complexity, whereas other metals showed a non-significant pattern (Figure 3I). Compared to 1 ppm, entropy was more broadly altered across nearly all metals, indicating more variable and highly unpredictable behavior (Figure 3J).

Overall, comparison across concentrations showed that entropy alterations became more prominent at 10 ppm, whereas thigmotaxis, meandering and fractal dimension exhibited metal-specific, non-monotonic responses. These findings indicate that behavioral endpoints differ in their sensitivity to increasing HM concentration rather than following a uniform dose–response pattern. Total distance travelled and entropy emerged as the most sensitive endpoints for distinguishing metal-specific neurobehavioral toxicity profiles in BSF larvae.

### 3.2. Behavioral Alterations of Black Soldier Fly Larvae Following Acute Rare Earth Element Exposure

**Figure 4:**
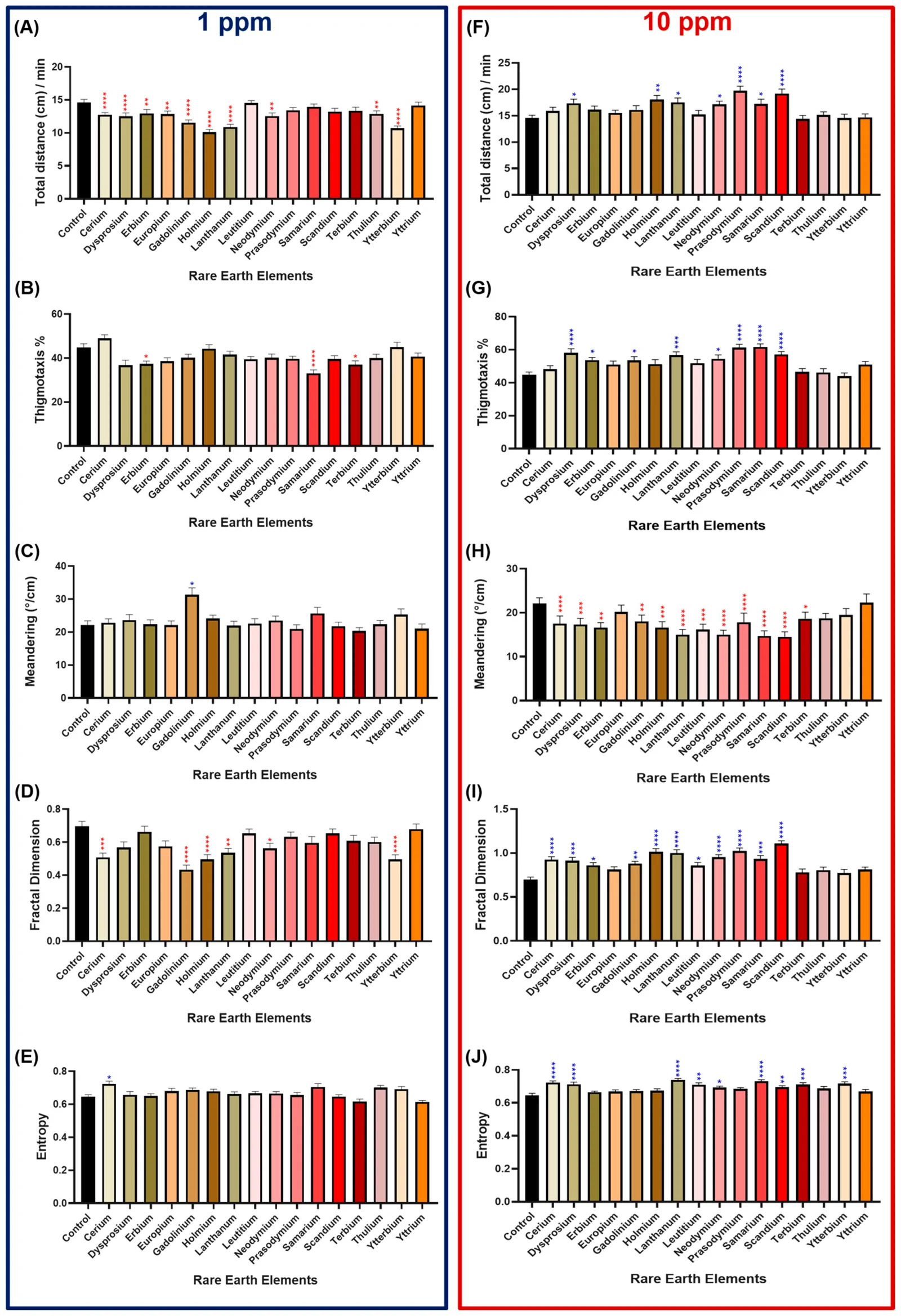
Black soldier fly (BSF) larvae were exposed to a panel of rare earth elements for 24 h, and behavioral responses were quantified using five endpoints. For the 1 ppm exposure group, measured parameters included (**A**) total distance travelled, (**B**) thigmotaxis, (**C**) meandering, (**D**) fractal dimension, and (**E**) entropy. The same behavioral endpoints were assessed for larvae exposed to 10 ppm rare earth elements: (**F**) total distance travelled, (**G**) thigmotaxis, (**H**) meandering, (**I**) fractal dimension, and (**J**) entropy. Data are presented as mean ± SEM and were analyzed using one-way ANOVA followed by Dunn’s multiple-comparison test. Statistical significance is indicated as * *p* < 0.05, ** *p* < 0.01, *** *p* < 0.001, and **** *p* < 0.0001 (*n* = 72).

A parallel behavioral screening assessed the acute effects of 16 rare earth elements (REEs) on Black Soldier Fly (BSF) larvae after 24 h exposure at 1 ppm using the same multi-endpoint framework (Figure 4A–E). In contrast to heavy metals, REEs produced a more selective and moderate response.

Locomotor suppression was the dominant effect, with total distance travelled significantly reduced by 10 of 16 REEs—cerium, dysprosium, erbium, europium, gadolinium, holmium, lanthanum, neodymium, thulium, and ytterbium, while others showed no significant change, indicating strong element-specific variability (Figure 4A). Behavioral organization was largely preserved at this concentration. Entropy remained stable across nearly all treatments, with cerium as the only REE causing a significant increase, suggesting minimal behavioral destabilization (Figure 4E). Fractal dimension analysis showed that several REEs reduced trajectory complexity, indicating mild spatial restriction without major disruption of movement organization (Figure 4D). Spatial and orientation behaviors were also minimally affected: meandering changed only with gadolinium (Figure 4C), while thigmotaxis was significantly altered by erbium, samarium, and terbium, with samarium showing the strongest effect (Figure 4B). Overall, 1 ppm REE exposure produced a fingerprint of selective locomotor suppression with largely preserved behavioral structure.

At 10 ppm (24 h; Figure 4F–J), larvae displayed a distinct high-concentration behavioral profile. In contrast to low concentration suppression, several REEs increased total distance travelled, including dysprosium, holmium, lanthanum, neodymium, praseodymium, scandium, and samarium, indicating locomotor excitation (Figure 4F). Thigmotaxis increased across multiple REEs, reflecting enhanced wall-following and altered spatial interaction (Figure 4G). Compared to 1 ppm, meandering was broadly reduced across most treatments, indicating straighter trajectories despite higher activity (Figure 4H). Fractal dimension increased for most REEs, reflecting more fragmented and less efficient movement paths (Figure 4I), while entropy also increased in a broad subset of elements, indicating higher behavioral variability and reduced predictability (Figure 4J).

Together, these results reveal a clear concentration-dependent biphasic response: low concentration (1 ppm) exposure induces selective locomotor suppression with preserved behavioral organization, whereas high concentration (10 ppm) exposure produces locomotor excitation accompanied by increased entropy, greater movement complexity, and altered spatial behavior. This non-monotonic pattern distinguishes REEs from heavy metals (Figure 3) and underscores the need for multi-concentration behavioral profiling to resolve distinct neurotoxic modes of action.

### 3.3. PCA and Hierarchical Clustering Reveal Heavy Metal and Rare Earth Elements Specific Behavioral Signatures

**Figure 5:**
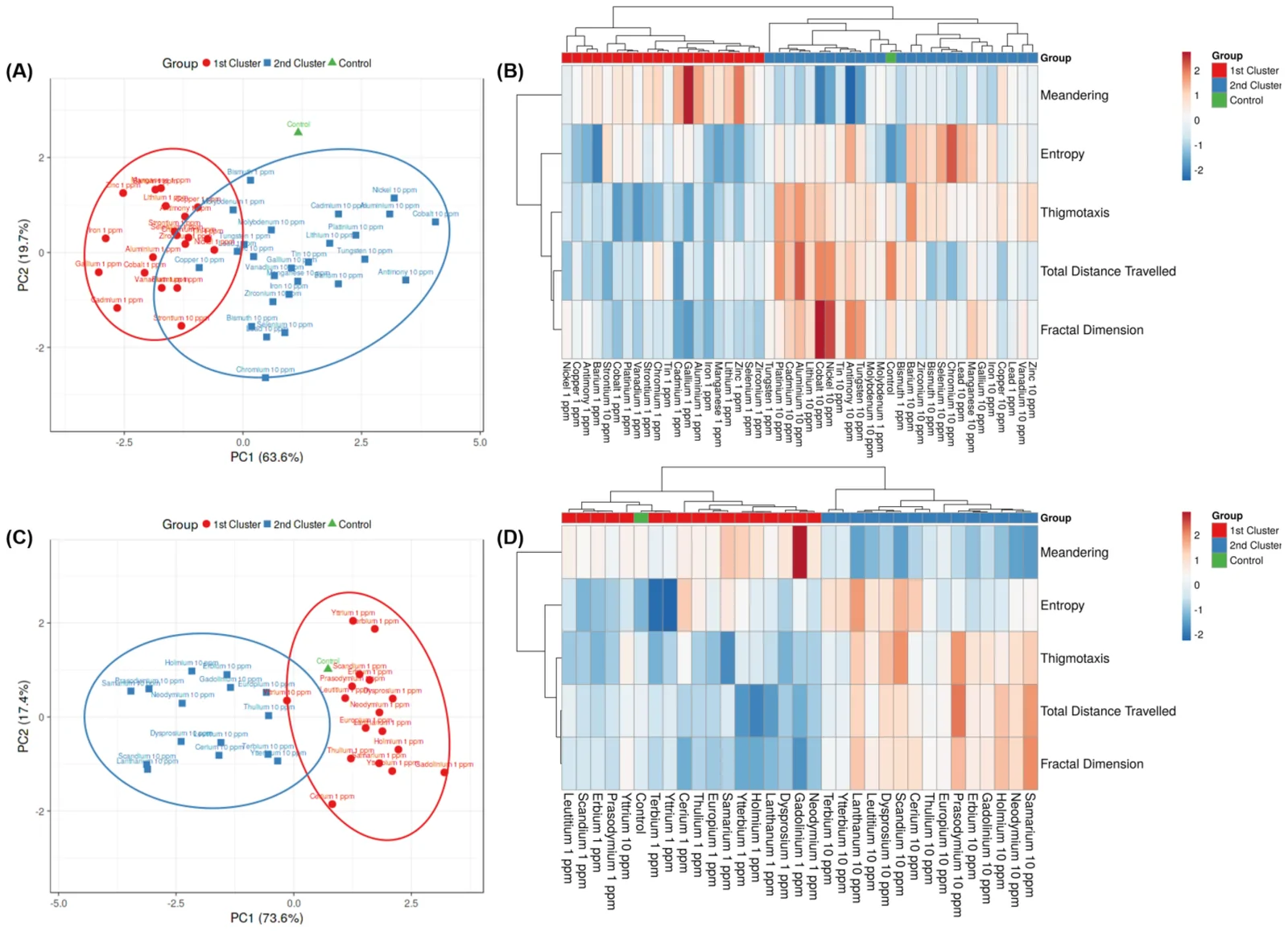
Differential neurobehavioral effects of heavy metals (HMs) and rare earth elements (REEs) in BSF larvae. (**A**) Principal Component Analysis (PCA) plot and (**B**) corresponding heatmap illustrate how 23 HMs alter locomotor activity across high and low concentrations. For HMs, principal components 1 and 2 explain 63.6% and 19.7% of variance (total 83.3%), capturing major patterns of behavioral variation. (**C**) PCA plot and (**D**) corresponding heatmap illustrate how 16 REEs alter locomotor activity across high and low concentrations. For REEs, principal components 1 and 2 explain 73.6% and 17.4% of variance (total 91%), capturing major patterns of behavioral variation. PCA plots cluster compounds based on locomotor response patterns, where closely grouped compounds share similar behavioral fingerprints. Heatmaps show activity changes relative to controls, with red indicating increased activity and light blue indicating decreased activity. Together, these analyses highlight compound-specific and concentration-dependent neurobehavioral toxicity patterns.

Figure 5 integrates principal component analysis (PCA) and hierarchical heatmap profiling to characterize the neurobehavioral effects of 23 heavy metals (HMs) and 16 rare earth elements in BSFL at low (1 ppm) and high (10 ppm) exposure concentrations. PCA (Figure 5A) showed clear segregation of treatment groups based on global behavioral signatures, with PC1 and PC2 explaining 83.3% of the total variance (Figure 5A,B). Separation was mainly driven by PC1, reflecting a concentration- and toxicity-dependent axis. Most 10 ppm exposures clustered on the positive side of PC1 (2nd cluster, blue), whereas most 1 ppm exposures grouped on the negative side (1st cluster, red), with controls clearly separated, indicating a distinct baseline behavioral phenotype. This segregation demonstrates that exposure concentration strongly influences the overall behavioral phenotype. However, individual behavioral endpoints did not all change monotonically with concentration, indicating that increasing HM concentration alters the composition of behavioral responses rather than uniformly amplifying every endpoint. The heatmap (Figure 5B) further defined the behavioral fingerprints underlying each PCA-derived cluster. The 1st cluster, dominated by 1 ppm exposures, showed anxiety-like and disorganized hypoactivity, characterized by reduced total distance travelled, increased thigmotaxis, elevated entropy, and decreased fractal dimension. This profile reflects suppressed locomotion, enhanced edge-seeking behavior, and simplified yet unpredictable movement trajectories, consistent with low-concentration neurobehavioral destabilization. In contrast, the second cluster, composed mainly of 10 ppm exposures, displayed severe motor suppression and highly unpredictable behavior, characterized by near-complete loss of locomotor activity, markedly increased entropy indicating disorganized and less predictable movement patterns, and minimal changes in thigmotaxis, suggesting that severe motor impairment overrides anxiety-related responses. Collectively, these PCA and heatmap analyses demonstrate that HM exposure induces distinct concentration-dependent neurobehavioral states in BSFL, ranging from stress-associated behavioral disorganization at low concentrations to extreme neurotoxicity and functional paralysis at high concentrations.

The neurobehavioral responses of BSFL to 16 rare earth elements (REEs) at 1 ppm and 10 ppm are summarized using combined principal component analysis and heatmap visualization (Figure 5C,D). Unlike heavy metals, REEs produced a distinct concentration-dependent behavioral landscape characterized by minimal disruption at low concentration and marked behavioral reorganization at high concentration. PCA (Figure 5C) revealed strong clustering by exposure concentration, with PC1 and PC2 accounting for 91.0% of the total variance. All 10 ppm REE treatments clustered tightly on one side of PC1, whereas 1 ppm treatments overlapped extensively with controls on the opposite side, indicating that low-concentration REE exposure maintained near-baseline behavioral profiles. Heatmap analysis (Figure 5D) further identified the behavioral features driving this separation and revealed a non-linear biphasic response pattern unique to REEs. The cluster containing control and 1 ppm exposures showed restricted suppression of locomotor output, with modest reductions in total distance travelled but minimal changes in entropy, thigmotaxis, meandering, or fractal dimension, indicating preserved behavioral organization despite reduced activity. In contrast, the 10 ppm cluster displayed behavioral excitation and reduced movement efficiency, characterized by elevated locomotor activity, increased entropy and fractal dimension reflecting fragmented and variable trajectories, reduced meandering, and increased thigmotaxis. Together, these findings demonstrate that REEs induce a concentration-dependent transition from mild selective locomotor suppression at low concentrations to a hyperexcitable and disorganized behavioral state at higher concentrations, distinct from the predominantly inhibitory effects observed for heavy metals.

### 3.4. PCA and Hierarchical Clustering Reveal Metallic Elements Behavioral Signatures

**Figure 6:**
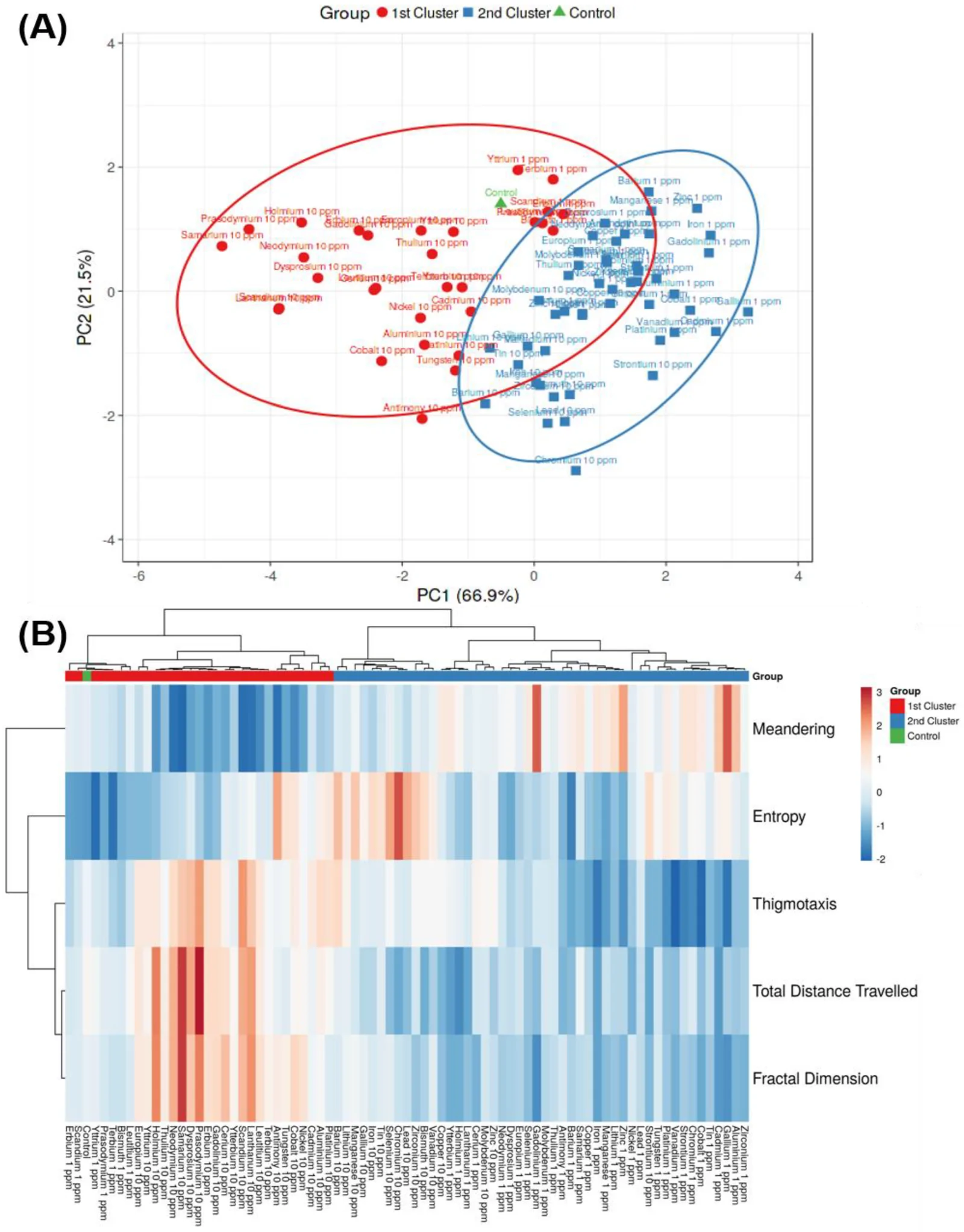
Differential neurobehavioral effects of all HMs and REEs tested in BSF larvae. (**A**) Principal Component Analysis (PCA) plot, and (**B**) corresponding heatmaps illustrate how 23 HMs and 16 REEs alter locomotor activity across low (1 ppm) and high (10 ppm) concentration. PCA plots act as behavioral maps, clustering chemicals based on locomotor response patterns. For REEs, principal components 1 and 2 explain 66.9% and 21.5% of variance (total 88.4%), capturing major patterns of behavioral variation. Chemicals positioned close together share similar behavioral fingerprints, suggesting related neurotoxic mechanisms, whereas those farther apart exhibit distinct profiles. Heatmaps display deviations from control activity, with red indicating increased activity and light blue indicating decreased activity. Hierarchical clustering further identifies compounds with shared locomotor effects, highlighting compound-specific and concentration-dependent neurobehavioral toxicity patterns.

Figure 6 integrates all metallic elements across both exposure concentrations into a unified phenomic space, enabling direct comparison of neurobehavioral toxicity across metal classes. The combined PCA (Figure 6A), including 78 treatment conditions, explained 88.4% of total variance, with PC1 representing a global toxicity axis separating highly disruptive from mild or near-control phenotypes. All 10 ppm heavy metal (HM) treatments and a subset of 10 ppm REEs clustered on the negative side of PC1, whereas most 1 ppm exposures from both classes clustered with controls on the positive side, indicating largely preserved behavioral states at low concentration. Partial overlap between classes was observed, as several high-concentration REEs clustered with high-concentration HMs, reflecting comparable levels of behavioral disruption.

Hierarchical clustering of the heatmap (Figure 6B) further resolved these overlaps into distinct behavioral fingerprints. Within the high-toxicity cluster, two subgroups emerged: one characterized by severe motor suppression and behavioral rigidity, with near-complete loss of locomotion and reduced entropy typical of high-concentration HMs, and another defined by hyperactive yet disorganized exploration, marked by elevated locomotion, entropy, and fractal dimension typical of high-concentration REEs. The low-toxicity cluster comprised anxiety-like disorganized hypoactivity induced by low-concentration HMs, and a selective locomotor suppression or minimal-effect profile including low-concentration REEs and controls.

Across Figure 5 and Figure 6, four reproducible neurobehavioral fingerprints were identified, shaped by both concentration and metal class. At 1 ppm, most HMs reduced locomotion, whereas only about half of REEs produced similar effects, indicating broader suppressive impact of HMs. At 10 ppm, HMs induced stronger reductions in entropy and more stereotyped behavior, whereas REEs primarily altered locomotor speed and movement complexity. Overall, HMs produced more profound and coherent neurobehavioral disruption than REEs at equivalent concentrations, highlighting behavioral phenomics as a tool to distinguish both toxicity severity and mechanistically distinct neurotoxic responses.

### 3.5. Cobalt Exposure Activates DNA Damage Response and Suppresses Neuroactive Signaling in BSFL

GSEA-based KEGG pathway enrichment analysis revealed distinct transcriptomic responses in BSFL following cobalt exposure compared with untreated controls. The 10 ppm cobalt treatment was selected for transcriptomic profiling because it produced one of the strongest behavioral phenotypes among the heavy metals, characterized by severe locomotor suppression and a high integrated toxicity ranking (Table S3). This analysis aimed to investigate the molecular basis of a representative high-toxicity HM phenotype rather than the mechanisms of all heavy metals. In the cobalt-versus-control comparison, 19 pathways reached significance (padj < 0.1), with NES values spanning from approximately −2.0 to +2.0 (Figure 6A). Among the most significantly upregulated pathways were DNA replication, cell cycle, cell cycle–yeast, meiosis–yeast, nucleotide excision repair, mismatch repair, and ribosome biogenesis in eukaryotes (NES > 1.5 for all), collectively indicating a robust activation of DNA damage sensing and repair programs. AMPK signaling and adipocytokine signaling were also positively enriched, suggesting perturbation of cellular energy homeostasis. Conversely, protein digestion and absorption, salivary secretion, and pancreatic secretion were among the most strongly downregulated pathways (NES ~ −2.0), indicating impairment of digestive physiology. Notably, the neuroactive ligand–receptor interaction pathway was significantly suppressed, consistent with the locomotor hypoactivity and behavioral disorganization documented in cobalt-exposed BSFL in the phenomic analyses (Figure 3 and Figure 4). Additional downregulated pathways included arginine and proline metabolism, glycosaminoglycan degradation, and beta-alanine metabolism, indicating broad metabolic disruption under cobalt stress.

### 3.6. Samarium Exposure Suppresses Xenobiotic Detoxification, Mitochondrial Function, and Synaptic Vesicle Cycling in BSFL

**Figure 7:**
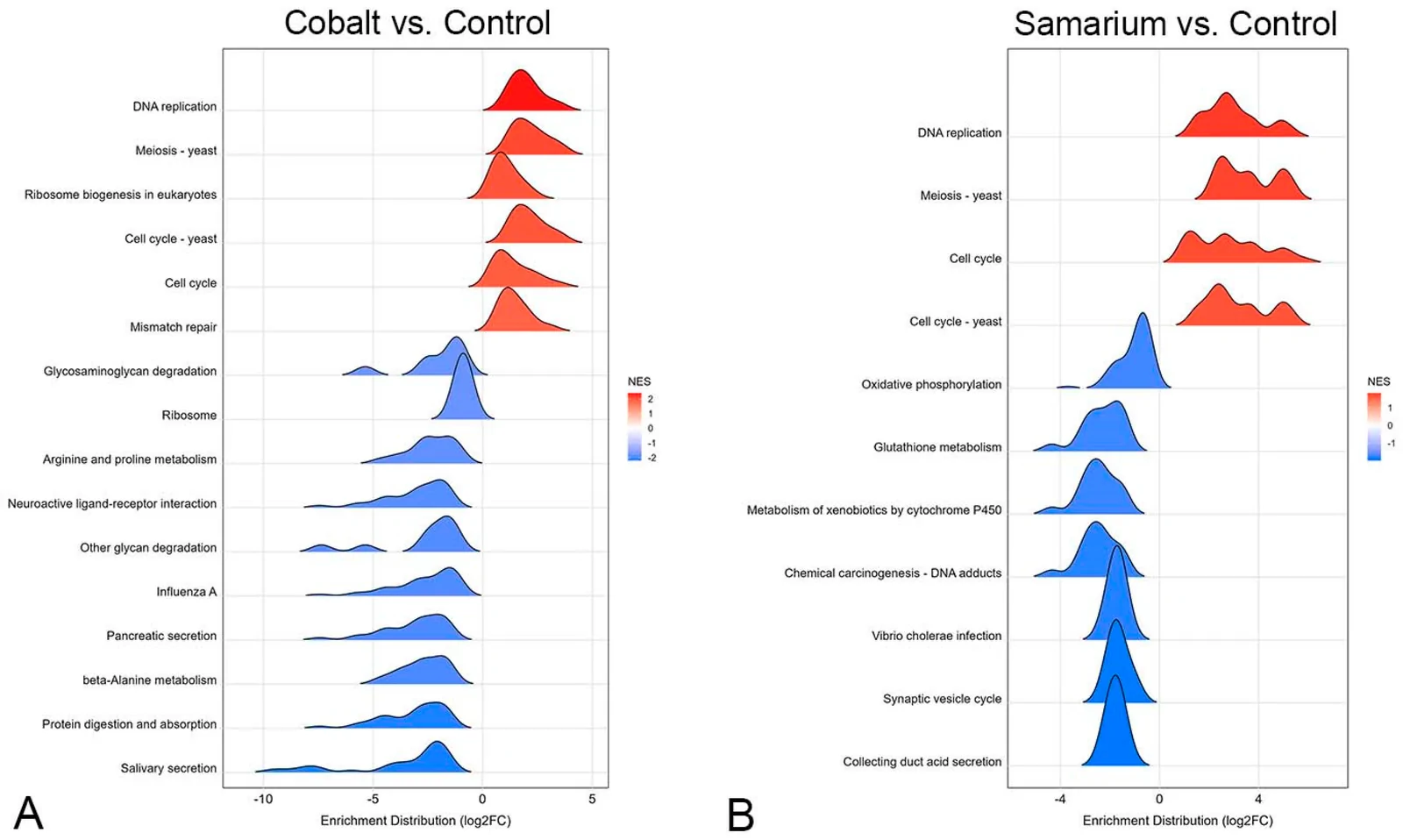
KEGG pathway gene set enrichment analysis (GSEA) of differentially expressed genes in *Hermetia illucens* larvae under cobalt and samarium exposure. Ridge plots displaying the enrichment distribution of leading-edge genes across significantly enriched KEGG pathways (FDR-adjusted *p* < 0.1) in (**A**) cobalt-treated versus control larvae and (**B**) samarium-treated versus control larvae. The *x*-axis represents the enrichment distribution of log2 fold-change values of leading-edge genes within each pathway. Pathways are ordered by normalized enrichment score (NES), with positively enriched pathways (NES > 0) shown in red and negatively enriched pathways (NES < 0) shown in blue. The top 10 positively and negatively enriched pathways are displayed for each comparison.

In the samarium-versus-control comparison, 14 pathways reached significance (padj < 0.1) with NES magnitudes generally restricted to ±1.0 (Figure 7B), indicating a comparatively attenuated but mechanistically distinct transcriptional response relative to cobalt. The 10 ppm samarium treatment was selected because it produced one of the most distinctive behavioral phenotypes among the REEs, characterized by pronounced thigmotaxis, locomotor excitation, and a high integrated toxicity ranking (Table S4). This analysis aimed to investigate the molecular basis of a representative REE behavioral phenotype rather than the mechanisms of all rare earth elements. Four pathways were positively enriched: DNA replication, cell cycle, cell cycle-yeast, and meiosis-yeast (NES ~ +1.0), suggesting a mild activation of cell proliferation-associated programs. In contrast, ten pathways were significantly downregulated. Among these, drug metabolism–cytochrome P450, metabolism of xenobiotics by cytochrome P450, and chemical carcinogenesis–DNA adducts were consistently suppressed. This suppression indicates a broad impairment of xenobiotic detoxification capacity. Oxidative phosphorylation and glutathione metabolism were also significantly downregulated, pointing to concurrent mitochondrial dysfunction and reduced antioxidant buffering.

Notably, the synaptic vesicle cycle pathway was significantly downregulated in samarium-exposed BSFL. This downregulation could be responsible for the dysregulation of synaptic transduction and provide a molecular basis for the pronounced elevation in thigmotaxis observed in samarium-exposed BSFL (Figure 4B), which was the strongest wall-associated behavioral response among all REEs tested. Furthermore, since beta-alanine is a precursor to the inhibitory neurotransmitter GABA, the downregulation of beta-alanine metabolism also supports our finding that samarium exposure induces locomotor excitation rather than motor inhibition (Figure 4F). Additional downregulated pathways included *Vibrio cholerae* infection, epithelial cell signaling in *Helicobacter pylori* infection, and collecting duct acid secretion; this likely reflects the suppression of conserved ion transport and epithelial signaling gene sets rather than infection-specific responses.

## 4. Discussion

Black soldier fly (*Hermetia illucens*) larvae are a promising model for high-throughput environmental toxicity assessment [55,56]. Our integrated behavioral phenomics and transcriptomics approach sensitively distinguished HM- and REE-induced neurobehavioral toxicity. Compared with vertebrate models, BSF larvae offer rapid growth, low cost, simple husbandry, high reproductive capacity, ecological relevance, and fewer ethical constraints [23]. They are easy to handle, require minimal infrastructure, and support large sample sizes for robust statistical analysis (*n* = 72, Figure 3 and Figure 4), enabling scalable toxicity screening. Prior studies have demonstrated their strong metal accumulation capacity, including for Cd, Pb, As, Cu, Hg, and Zn, depending on exposure conditions. Cadmium retention can reach up to 93% [57], whereas arsenic and chromium may be partially eliminated through frass excretion, indicating intrinsic detoxification mechanisms [58]. Heavy metal exposure affects growth, survival, oxidative stress, metabolism, and gut microbiota, while behavioral effects remain underexplored [23]. Integrating behavioral and transcriptomic analyses enables behavioral–molecular characterization of metal-induced alterations (Figure 3, Figure 4, Figure 5, Figure 6 and Figure 7) [59]. Despite these advances, standardized behavioral frameworks remain limited.

To address this gap, and based on the intrinsic behavioral characteristics of BSF larvae, we established a high-throughput and standardized experimental pipeline to quantify locomotor behavior for environmental toxicity assessment under metallic toxicant exposure. The workflow consists of four stages: (1) Acute toxicants exposure using immersion method, (2) controlled behavior recording, (3) automated motion tracking, and (4) extraction of quantitative behavioral endpoints. Behavioral recording used a custom transparent acrylic arena with 24 isolated compartments and the ZebraBox system [24,60], enabling simultaneous multi-larvae observation under controlled conditions with consistent illumination and spatial separation [24]. For motion tracking, idTracker, ToxTrac, and UMATracker were compared, with UMATracker showing the most stable performance for BSFL by tracking larval centroids [24,61]. Given the relatively stable cylindrical morphology of BSFL, centroid-based tracking provided reliable overall movement quantification, although fine-scale body dynamics were not captured [62]. Despite this limitation, UMATracker offered the best balance between accuracy and throughput for large-scale screening. Finally, tracked data were converted into quantitative behavioral endpoints for objective assessment of contaminant-induced locomotor changes. While AI-based full-body tracking may improve resolution, this pipeline provides a robust and scalable framework for high-throughput BSFL behavioral toxicology under laboratory conditions.

Building on this framework, this study positions BSF larvae as scalable bioindicators for HM and REE toxicity through integrated behavioral–molecular analysis and links their responses to bioremediation and circular economy applications [63,64]. Collectively, BSF larvae represent a low-cost, high-throughput platform for environmental monitoring and metallic pollutant assessment [23,65].

We used optimized behavioral phenotyping to assess 23 HMs and 16 REEs at 1 and 10 ppm, revealing concentration-related changes in neurobehavioral responses that varied across behavioral endpoints. At 1 ppm, HMs caused broader behavioral impairment than REEs, including reduced locomotion, increased thigmotaxis, and decreased fractal dimension and entropy (Figure 3). In contrast, REEs produced milder and more selective effects, indicating distinct toxicity profiles rather than a common stress response (Figure 4). Among HM-induced alterations, reduced total distance traveled emerged as the most sensitive and consistent indicator of early toxicity. Increased thigmotaxis, together with altered entropy and fractal dimension, further indicates disrupted movement organization and stress-related edge-seeking behavior [66,67]. By contrast, meandering was relatively resistant at low HM exposure, suggesting that directional stability is less sensitive than overall activity or movement complexity [68]. This pattern is consistent with a hierarchical sensitivity of behavioral endpoints, where activity-related measures respond earlier than orientation and path-structure metrics under sublethal exposure [69]. Overall, low-concentration HM toxicity is characterized by hypoactivity combined with behavioral disorganization. REE exposure at 1 ppm showed a distinct pattern (Figure 3), with locomotor suppression observed only in selected elements and generally weaker and less uniform effects compared to HMs [70]. Entropy remained largely unchanged, and only a subset of REEs affected thigmotaxis or meandering, indicating more element-specific and selective toxicity.

Importantly, both HMs and REEs exhibited behavioral changes across the two tested concentrations, but the response patterns differed markedly among behavioral endpoints. For HMs, locomotor suppression and entropy disruption generally became pronounced at 10 ppm, whereas thigmotaxis, meandering, and fractal dimension did not follow a consistent monotonic concentration-response relationship. Instead, these endpoints showed metal-specific non-monotonic responses, with some metals producing stronger effects at 1 ppm than at 10 ppm and others showing the opposite pattern. These findings indicate that HM-induced neurobehavioral toxicity is endpoint-dependent rather than uniformly increasing with concentration [71]. In contrast, REEs displayed a biphasic concentration-dependent behavioral profile. At 1 ppm, several REEs induced selective locomotor suppression with reduced fractal dimension, while thigmotaxis, meandering, and entropy remained largely unchanged. At 10 ppm, REEs shifted toward locomotor excitation, accompanied by increased thigmotaxis, fractal dimension, and entropy, and reduced meandering, indicating more fragmented and unpredictable movement patterns [72,73]. PCA further confirmed concentration-dependent separation, identifying concentration as a major driver of phenotypic divergence in both pollutant classes. These behavioral patterns suggest partially overlapping but distinct toxic mechanisms [74,75]. Increasing HM concentration shifted the overall neurobehavioral profile rather than uniformly amplifying every behavioral endpoint, highlighting the endpoint-specific nature of HM neurotoxicity. In contrast, REEs may disrupt locomotor regulation through more complex mechanisms involving calcium signaling, synaptic function, or detoxification pathways [76]. Overall, these findings highlight the importance of multi-endpoint, multi-concentration behavioral analyses for ecological risk assessment. Notably, no mortality was observed at 1 or 10 ppm, indicating that survival-based endpoints would miss substantial sublethal impairments [77,78]. Therefore, behavioral phenomics provides a more sensitive and informative framework for assessing sublethal toxicity in BSF larvae and enhances their applicability as a model system for environmental risk assessment.

To further elucidate the molecular basis underlying the distinct behavioral phenotypes observed during phenomic screening, GSEA-based KEGG transcriptomic analyses were performed using cobalt (Co) and samarium (Sm) as representative toxicants for heavy metals (HMs) and rare earth elements (REEs), respectively. These compounds were selected based on their prominent and mechanistically distinct behavioral effects in BSFL, as well as their integrated toxicity rankings derived from hierarchical clustering-associated numeric differences (Tables S3 and S4). The analyses revealed that cobalt and samarium induce toxicity through fundamentally different molecular pathways, providing mechanistic insight into the divergent behavioral fingerprints observed in the phenomic analyses.

The dominant transcriptomic signature of cobalt exposure was the coordinated activation of DNA damage response pathways. This finding is mechanistically consistent with the toxicological effects of cobalt, which generates hydroxyl radicals through Fenton-like redox cycling, producing oxidative DNA lesions that necessitate the concurrent induction of both nucleotide excision repair and mismatch repair [79]. Additionally, cobalt stabilizes HIF-1α and induces a pseudo-hypoxic metabolic state that elevates the AMP:ATP ratio and triggers AMPK activation [80,81]. In contrast, the samarium transcriptomic response was defined by the suppression of xenobiotic detoxification and mitochondrial function rather than genotoxic stress. The downregulation of drug metabolism pathways sharing overlapping CYP enzymes indicates a broad impairment of phase I detoxification capacity. CYP monooxygenases constitute the primary xenobiotic defense system in insects [82], and their suppression by samarium exposure would render BSFL disproportionately vulnerable to co-occurring environmental pollutants. This finding carries particular ecological relevance given the well-documented co-occurrence of REEs and heavy metals in mining-impacted soils and industrial effluents [83]. The concurrent downregulation of oxidative phosphorylation and glutathione metabolism in BSFL further implicates mitochondrial dysfunction and compromised antioxidant defense as secondary toxicity axes. As a trivalent lanthanide, Sm^3+^ closely mimics Ca^2+^ at biological binding sites [84] and, like other lanthanide cations, inhibits active Ca^2+^ transport across biological membranes [85], thereby uncoupling Ca^2+^-dependent stimulation of TCA cycle dehydrogenases and reducing ATP synthesis. Perhaps the most behaviorally informative finding in samarium-exposed BSFL was the downregulation of the synaptic vesicle cycle pathway. Sm^3+^ displacement of Ca^2+^ at presynaptic active zones would impair neurotransmitter release. This presynaptic mechanism explains why samarium produced the strongest thigmotaxis elevation of any REE at 1 ppm without causing the global locomotor collapse characteristic of cobalt exposure.

Collectively, these findings establish that heavy metals and REEs carry distinct ecotoxicological risk profiles beyond their comparative behavioral potency. Cobalt poses a direct genotoxic and broad neurotoxic hazard, well-captured by conventional behavioral and mutagenicity endpoints. In contrast, samarium’s primary toxicological actions are likely mediated mechanistically through CYP450 detoxification impairment and presynaptic Ca^2+^ disruption. Nevertheless, these mechanistic interpretations should be viewed as representative case studies rather than universal signatures of all HMs or REEs. Both metal classes comprise chemically diverse elements with distinct physicochemical properties, oxidation states, and biological targets, resulting in considerable variability in their mechanisms of toxicity. Consequently, other heavy metals or rare earth elements may engage different molecular pathways despite producing partially overlapping behavioral phenotypes. Future transcriptomic analyses encompassing a broader range of metals will be necessary to define both shared and metal-specific mechanisms of toxicity. Our results underscore the value of integrating behavioral phenomics with transcriptomic pathway analysis as a comprehensive platform for environmental metal risk assessment, and establish BSFL as a sensitive and mechanistically informative bioindicator for both HM and REE contamination.

## 5. Conclusions

This study establishes Black Soldier Fly larvae (BSFL) as a sensitive, high-throughput invertebrate model for side-by-side comparison of the sublethal neurobehavioral toxicity of 23 heavy metals (metalloids) (HMs) and 16 rare earth elements (REEs) across environmentally relevant concentrations. Using a video-tracking-based phenomics pipeline, integrating distance traveled, entropy, fractal dimension, meandering, and thigmotaxis with multivariate clustering and toxicity ranking, the work reveals four reproducible behavioral fingerprints shaped by both metal class and concentration.

**Figure 8:**
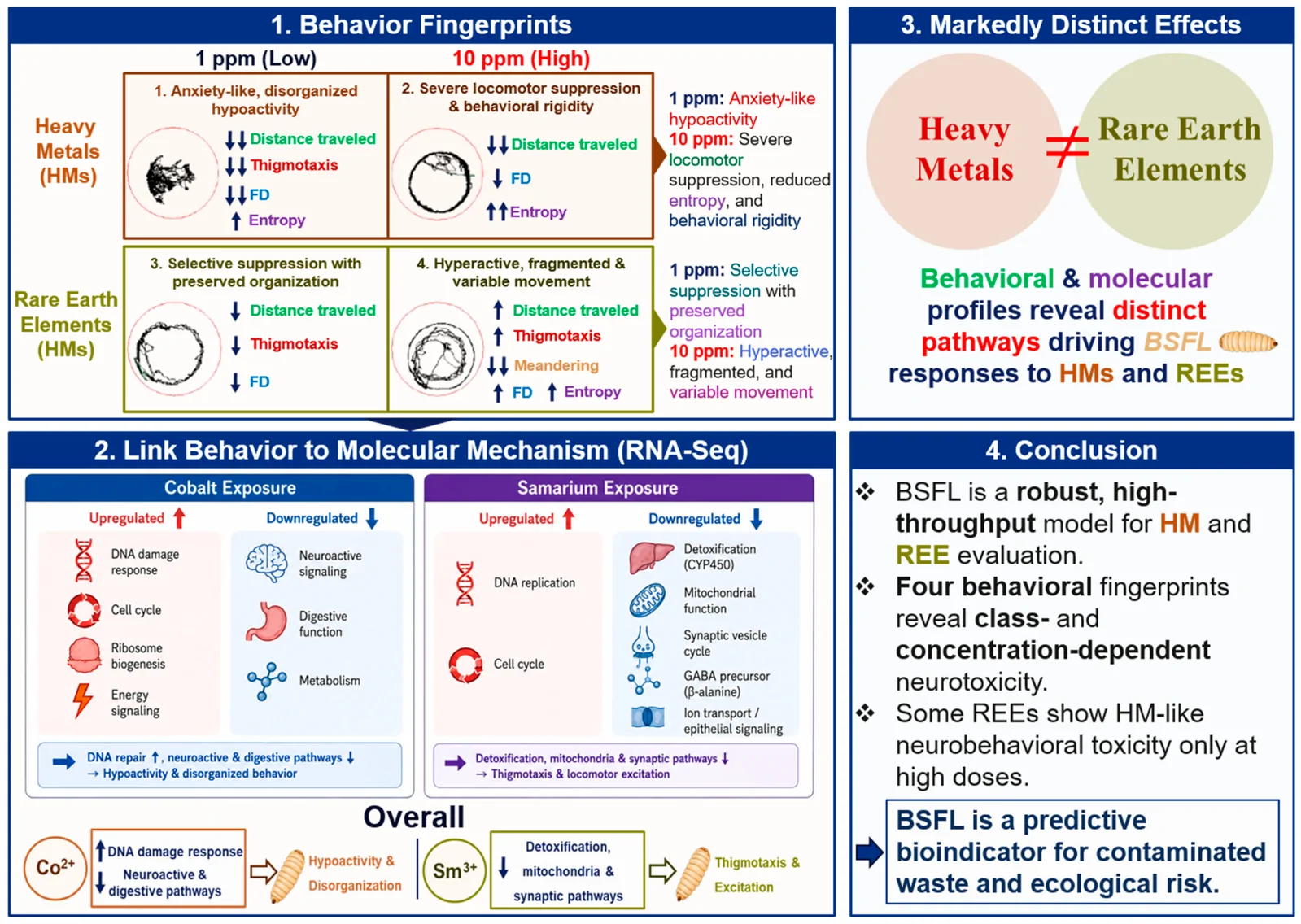
Summary of integrated behavioral phenomics and transcriptomic responses of Black Soldier Fly larvae (BSFL) following heavy metal (HM) and rare earth element (REE) exposure. Behavioral profiling identified four concentration- and class-dependent neurobehavioral fingerprints: anxiety-like disorganized hypoactivity, and severe locomotor suppression induced by HMs, and selective locomotor suppression with preserved organization and hyperactive fragmented movement induced by REEs. Transcriptomic analyses revealed distinct molecular mechanisms, with cobalt activating DNA damage repair, energy stress, and neuroactive pathway disruption, while samarium suppressed xenobiotic detoxification, mitochondrial function, and synaptic vesicle cycling. Together, the findings establish BSFL as a robust, high-throughput bioindicator for ecological risk assessment of metallic pollutants and contaminated waste systems (the figure was created by using biorender.com).

HMs generally induced stronger and more consistent neurobehavioral disruption, characterized by locomotor suppression, increased thigmotaxis, reduced movement complexity, and behavioral rigidity, particularly at high concentrations. In contrast, REEs exhibited more selective and biphasic effects, transitioning from mild locomotor suppression at low concentration to hyperactive and disorganized behavior at higher concentration. Combined phenomic analyses resolved four reproducible behavioral fingerprints, demonstrating that toxicity is shaped by both metal class and exposure level. Transcriptomic profiling of cobalt and samarium, selected as representative case-study compounds based on their distinctive behavioral phenotypes and toxicity rankings, revealed mechanistically distinct molecular responses. Cobalt, representing a high-toxicity HM phenotype, primarily activated DNA damage repair and energy stress pathways, whereas samarium, representing a characteristic REE phenotype, suppressed xenobiotic detoxification, mitochondrial function, and synaptic vesicle cycling (Figure 8). Together with the behavioral phenomic analyses, these findings demonstrate contrasting molecular pathways underlying representative HM and REE exposures, providing a systems-level understanding of metal-induced toxicity in BSFL. However, because cobalt and samarium were selected as representative case studies, these molecular responses should be interpreted as mechanistic examples rather than universal signatures of all heavy metals or rare earth elements. Overall, this study provides the first large-scale integrated behavioral–-transcriptomic comparison of HMs and REEs in BSFL, addressing a key knowledge gap in emerging metallic pollutants and establishing BSFL phenomics coupled with molecular profiling as a scalable, mechanistically informative framework for ecological risk assessment, environmental monitoring, and evaluation of metal-contaminated waste bioconversion systems.

## Data Availability

Original videos have been deposited to Zenodo database (https://zenodo.org/records/18143985, accessed on 18 January 2026). RNA-seq data (FASTQ files) have been deposited in the NCBI Sequence Read Archive (SRA) under BioProject accession number PRJNA 1467192.

## Supplementary Materials

The following supporting information can be downloaded at: https://www.mdpi.com/article/10.3390/toxics14080729/s1, Table S1: Heavy metal chloride compounds with CAS numbers used in BSF larvae toxicity assays; Table S2: Rare earth element chloride compounds with CAS numbers used in BSF larvae toxicity assays; Table S3: Integrated toxicity ranking of heavy metals based on hierarchical clustering-derived numeric differences; Table S4: Integrated toxicity ranking of rare earth elements based on hierarchical clustering-derived numeric differences.
