# Supplementary material for: Comparing the Behavioral Impacts of Heavy Metals and Rare Earth Elements on Black Soldier Fly (Hermetia illucens) Larvae
Source: Toxics. 2026 Aug 17;14(8):729. doi: 10.3390/toxics14080729 (PMC13517398; doi:10.3390/toxics14080729)
Supplement: Supplementary file 1 [file toxics-14-00729-s001.zip › toxics-4469279-supplementary.pdf]

Article

Comparing the Behavioral Impacts of Heavy Metals and Rare Earth Elements on Black Soldier Fly (*Hermetia illucens*) Larvae

Muhammad Baqir Khan [1], Minh-Quan Tran [1,2], Petrus Siregar [1], Szu-Chieh Wang [3], Ming-Der Lin [3,4,*] and Chung-Der Hsiao [1,2,5,6,7,*]

[1] Department of Biosciences, Chung Yuan Christian University, No. 200, Zhongbei Rd., Zhongli Dist., Taoyuan 320314, Taiwan; baqirkhanmmc@gmail.com (M.B.K.); minhquan0141@gmail.com (M.-Q.T.); siregar.petrus27@gmail.com (P.S.)

[2] Department of Biomedical Engineering, Chung Yuan Christian University, No. 200, Zhongbei Rd., Zhongli Dist., Taoyuan 320314, Taiwan

[3] Department of Molecular Biology and Human Genetics, Tzu Chi University, Hualien 970374, Taiwan; scwang2023@gmail.com

[4] Center for Herbal Medicine and Natural Products Research, Tzu Chi University, Hualien 970374, Taiwan

[5] Department of Biotechnology, Kaohsiung Medical University, Kaohsiung City 80708, Taiwan

[6] Department of Chemistry, Chung Yuan Christian University, Taoyuan 320314, Taiwan

[7] Research Center of Aquatic Toxicology and Pharmacology, Chung Yuan Christian University, Taoyuan 320314, Taiwan

* Correspondence: mingder@gms.tcu.edu.tw (M.-D.L.); cdhsiao@cycu.edu.tw (C.-D.H.)

Table S1. Heavy metal chloride compounds with CAS numbers used in BSF larvae toxicity assays.

**Heavy Metal**	**Chloride Form**	**CAS Number**
Aluminum	Aluminum chloride ($AlCl_3$)	7446-70-0
Antimony	Antimony trichloride ($SbCl_3$)	10025-91-9
Barium	Barium chloride ($BaCl_2$)	10361-37-2
Bismuth	Bismuth trichloride ($BiCl_3$)	7787-60-2
Cadmium	Cadmium chloride ($CdCl_2$)	10108-64-2
Chromium	Chromium (III) chloride ($CrCl_3$)	10025-73-7
Cobalt	Cobalt (II) chloride ($CoCl_2$)	7646-79-9
Copper	Copper (II) chloride ($CuCl_2$)	7447-39-4
Gallium	Gallium (III) chloride ($GaCl_3$)	13450-90-3
Iron	Iron (III) chloride ($FeCl_3$)	7705-08-0
Lead	Lead (II) chloride ($PbCl_2$)	7758-95-4
Lithium	Lithium chloride (LiCl)	7447-41-8
Manganese	Manganese (II) chloride ($MnCl_2$)	7773-01-5
Molybdenum	Molybdenum (V) chloride ($MoCl_5$)	10241-05-1
Nickel	Nickel (II) chloride ($NiCl_2$)	7718-54-9
Platinum	Platinum (IV) chloride ($PtCl_4$)	13454-96-1
Selenium	Selenium (IV) chloride ($SeCl_4$)	10026-03-6
Strontium	Strontium chloride ($SrCl_2$)	10476-85-4
Tin	Tin (II) chloride ($SnCl_2$)	7772-99-8
Tungsten	Tungsten (VI) chloride (WCl_6)	13283-01-7
Vanadium	Vanadium (III) chloride (VCl_3)	7718-98-1
Zinc	Zinc chloride ($ZnCl_2$)	7646-85-7
Zirconium	Zirconium (IV) chloride ($ZrCl_4$)	10026-11-6

Table S2. Rare earth element chloride compounds with CAS numbers used in BSF larvae toxicity assays.

**Rare earth elements**	**Chloride Form**	**CAS Number**
Cerium	Cerium (III) chloride heptahydrate	18618-55-8
Dysprosium	Dysprosium (III) chloride hexahydrate	15059-52-6
Erbium	Erbium (III) chloride hexahydrate	10025-75-9
Europium	Europium (III) chloride hexahydrate	13759-92-7
Gadolinium	Gadolinium (III) Chloride Hexahydrate	13450-84-5
Holmium	Holmium (III) Chloride hexahydrate	14914-84-2
Lanthanum	Lanthanum (III) chloride hexahydrate	17272-45-6

Lutetium	Lutetium (III) chloride hexahydrate	15230-79-2
Neodymium	Neodymium (III) chloride hexahydrate	13477-89-9
Praseodymium	Praseodymium (III) chloride	10361-79-2
Terbium	Terbium (III) chloride hexahydrate	13798-24-8
Thulium	Thulium (III) chloride hexahydrate	1331-74-4
Samarium	Samarium (III) Chloride hexahydrate	13465-55-9
Scandium	Scandium (III) chloride	10361-84-9
Ytterbium	Ytterbium (III) chloride	10361-91-8
Yttrium	Yttrium (III) chloride hexahydrate	10025-94-2

Table S3. Integrated toxicity ranking of heavy metals based on hierarchical clustering-derived numeric differences (follow ranking order).

Compounds	Behavioral Numeric Differences					Total Absolute Numeric Differences
Cobalt 10 ppm	2.1	0.64	1.27	1.9	2.75	**8.66**
Antimony 10 ppm	2.42	1.53	0.66	1.18	1.9	7.69
Aluminium 10 ppm	1.07	0.42	1.73	2.22	1.32	6.76
Iron 1 ppm	0.95	1.33	1.48	1.46	1.41	6.63
Nickel 10 ppm	1.59	0.02	0.82	1.86	2.34	6.63
Gallium 1 ppm	2.67	0.31	1.55	0.37	1.7	6.6
Cadmium 1 ppm	1.51	0.24	0.92	1.74	1.52	5.93
Zinc 1 ppm	2.02	1.51	1.15	0.17	0.91	5.76
Tungsten 10 ppm	1.66	0.73	0.75	0.71	1.69	5.54
Platinum 10 ppm	0.95	0.54	1.64	1.8	0.42	5.35
Barium 10 ppm	0.39	1.27	1.81	0.97	0.38	4.82
Barium 1 ppm	0.64	2.06	0.85	0.84	0.39	4.78
Cadmium 10 ppm	0.64	0.18	1.38	1.21	1.11	4.52
Chromium 10 ppm	0.57	2.27	0.3	1.31	0.05	4.5
Cobalt 1 ppm	0.68	0.04	1.7	0.73	1.2	4.35
Manganese 1 ppm	0.78	1.6	1.02	0.05	0.84	4.29
Strontium 10 ppm	0.26	0.86	0.69	1.46	0.89	4.16
Vanadium 1 ppm	0.11	0.35	1.79	0.82	1.07	4.14
Lithium 10 ppm	0.48	0.46	1.12	1.04	0.96	4.06
Selenium 10 ppm	0.15	1.53	0.84	0.86	0.67	4.05
Copper 10 ppm	0.76	0.47	0.93	1.1	0.76	4.02
Aluminium 1 ppm	1.57	0.27	0.54	0.6	1.01	3.99
Lithium 1 ppm	1.16	1.25	0.59	0.14	0.69	3.83
Chromium 1 ppm	0.99	0.13	1.29	0.7	0.69	3.8
Bismuth 10 ppm	0.4	0.93	0.88	1.21	0.37	3.79
Antimony 1 ppm	0.45	1.41	0.6	1.02	0.21	3.69
Platinum 1 ppm	0.64	0.49	1.22	0.73	0.61	3.69
Lead 10 ppm	0.82	1.48	0.24	0.87	0.14	3.55
Bismuth 1 ppm	0.92	1.43	0.4	0.71	0.03	3.49
Selenium 1 ppm	0.76	0.88	0.18	0.59	0.83	3.24
Tin 10 ppm	0.66	0.77	0.42	0.82	0.57	3.24

Zirconium 10 ppm	0.22	1.17	0.93	0.25	0.67	3.24
Strontium 1 ppm	0.9	0.38	1.38	0.08	0.49	3.23
Manganese 10 ppm	0.08	1.2	0.19	0.43	1.15	3.05
Nickel 1 ppm	0.24	0.7	0.44	1.13	0.28	2.79
Zirconium 1 ppm	0.17	0.6	0.51	0.39	1.08	2.75
Iron 10 ppm	0.93	0.91	0.36	0.15	0.28	2.63
Gallium 10 ppm	0.8	0.31	0.43	0.2	0.8	2.54
Copper 1 ppm	0.13	1.18	0.92	0.19	0.09	2.51
Vanadium 10 ppm	0.04	0.38	0.88	0.34	0.5	2.14
Zinc 10 ppm	0.1	0.28	1.01	0.43	0.09	1.91
Molybdenum 10 ppm	0.05	0.38	0.96	0.41	0.1	1.9
Molybdenum 1 ppm	0.19	0.84	0.12	0.45	0.24	1.84
Tin 1 ppm	0	0.43	0.64	0.05	0.58	1.7
Tungsten 1 ppm	0.2	0.02	0.66	0.2	0.29	1.37
Lead 1 ppm	0.18	0.07	0.2	0	0.21	0.66

Table S4. Integrated toxicity ranking of rare earth elements based on hierarchical clustering-derived numeric differences (follow ranking order).

**Compounds**	**Behavioral Numeric Differences**					**Total Absolute Numeric Differences**
Samarium 10 ppm	1.57	0.42	1.37	1.95	2.02	**7.33**
Lanthanum 10 ppm	1.44	1.89	1.33	1.23	1.43	7.32
Scandium 10 ppm	1.51	1.61	1.96	1.12	1.07	7.27
Gadolinium 1 ppm	2.94	0.15	0.81	1.27	1.68	6.85
Praseodymium 10 ppm	0.68	0.08	1.9	2.17	1.56	6.39
Dysprosium 10 ppm	0.82	1	1.5	1.17	0.95	5.44
Neodymium 10 ppm	1.44	0.33	1.04	1.08	1.17	5.06
Samarium 1 ppm	1.41	0.78	1.73	0.27	0.79	4.98
Holmium 10 ppm	1.01	0.28	0.62	1.46	1.5	4.87
Ytterbium 1 ppm	1.32	0.34	0.19	1.62	1.34	4.81
Dysprosium 1 ppm	0.87	0.83	1.26	0.86	0.94	4.76
Holmium 1 ppm	1	0.14	0.29	1.86	1.34	4.63
Terbium 1 ppm	0	2.13	1.22	0.52	0.72	4.59
Cerium 1 ppm	0.65	1.38	0.32	0.76	1.27	4.38
Neodymium 1 ppm	0.83	0.56	0.81	0.85	0.97	4.02
Lanthanum 1 ppm	0.42	0.28	0.63	1.55	1.12	4
Cerium 10 ppm	0.77	1.34	0.22	0.56	1.02	3.91
Erbium 1 ppm	0.53	1.04	1.18	0.68	0.42	3.85
Erbium 10 ppm	1.01	0.6	0.93	0.65	0.66	3.85
Yttrium 1 ppm	0.18	2.25	0.75	0.18	0.34	3.7
Lutetium 10 ppm	1.13	0.92	0.69	0.27	0.64	3.65
Thulium 1 ppm	0.53	0.63	0.84	0.71	0.76	3.47
Scandium 1 ppm	0.36	1.16	0.89	0.57	0.47	3.45
Gadolinium 10 ppm	0.63	0.36	0.92	0.65	0.77	3.33

Europium 1 ppm	0.46	0.06	1.03	0.73	0.9	3.18
Praseodymium 1 ppm	0.15	0.85	0.88	0.49	0.59	2.96
Lutetium 1 ppm	0.59	0.53	0.91	0.03	0.48	2.54
Yttrium 10 ppm	0.51	0.45	0.58	0.06	0.4	2
Ytterbium 10 ppm	0.25	1.14	0.33	0	0.19	1.91
Europium 10 ppm	0.05	0.45	0.58	0.4	0.4	1.88
Terbium 10 ppm	0.47	0.97	0.02	0.08	0.22	1.76
Thulium 10 ppm	0.44	0.19	0.04	0.23	0.36	1.26
